# Piperacillin/Tazobactam (ZOSYN)

**DOI:** 10.1155/S1064744996000506

**Published:** 1996

**Authors:** Stephanie M. Culver, Mark G. Martens

**Affiliations:** Department of Obstetrics and Gynecology Hennepin County Medical Center 701 Park Avenue South Minneapolis MN 55415-1829 USA


Infectious Diseases in Obstetrics and Gynecology 4:258–262 (1997)
© 1997 Wiley-Liss, Inc.

### Piperacillin/Tazobactam (ZOSYN)

**Stephanie M. Culver and Mark G. Martens**
*Department of Obstetrics and Gynecology, Hennepin County Medical Center, Minneapolis, MN*

Piperacillin/tazobactam (ZOSYN, Lederle Laboratories, Pearl River, NY) is the most recently approved combination of a β-lactam antimicrobial agent with an inhibitor of bacterial β-lactamases. In vitro and clinical studies have demonstrated efficacy in polymicrobial infections such as obstetric, gynecologic, and soft-tissue infections. Piperacillin/tazobactam appears to be an appropriate drug for the empiric treatment of gynecologic infections because of its broad spectrum, good penetration of tissues, and minimal side-effect profile. Multiple studies have demonstrated that piperacillin/tazobactam, compared with "gold standard" treatment regimens, is an equally effective and safe alternative. It may also prove to be more cost effective, based on early reports of broad microbiologic coverage and high cure rates.

### STRUCTURE AND DERIVATION

Piperacillin is a semisynthetic ureidopenicillin with an extended spectrum of activity. Piperacillin sodium is derived from D(−)-α-aminobenzylpenicillin. The chemical name is sodium *(2S, 5R, 6R)*-*6*-[(*R*)-2-(4-ethyl-2,3-dioxo-piperazine-carboxyamido)-2-phenylacetamido]-3,3-dimethyl-7-oxo-4-thia-1-azabicyclo [3.2.1]-heptane-2-carboxylate.[1]

Tazobactam sodium is a triazolymethyl penicillanic-acid sulfone derivative. The triazole ring facilitates the binding of tazobactam to β-lactamases. The chemical name is sodium (*2S, 3S, 5R*)-3-methyl-7-oxo-3-(*1H,2,3*-triazol-1-ylmethyl)-4-thia-1-azabicyclo-[3.2.0]-heptane-2-carboxylate-4, 4-dioxide.

Both structures are highly water soluble, with pKas of 2.1 and 4.14, respectively.[2]

### MECHANISM OF ACTION

All penicillins share a basic structure of a thiazolidine ring connected to a β-lactam ring with an attached side chain. The properties of penicillin are affected by altering the side chain, thus forming a variety of different penicillin compounds. Penicillins essentially have the same bactericidal mechanism, interfering with the synthesis of the peptidoglycan component of the cell wall. In recent years, β-lactam drugs have been combined with β-lactam inhibitors. β-lactam inhibitors have the ability to bind irreversibly to β-lactamase enzymes, thereby increasing the antimicrobial activity of several β-lactam antibiotics. Although tazobactam has little intrinsic antimicrobial activity, it binds covalently to a clinically important number of plasmids and chromosomally mediated β-lactamases. It has enhanced piperacillin's bactericidal activity.[3] Tazobactam provides an added advantage over other β-lactam inhibitors in that it exhibits minimal induction of class-1, chromosomally mediated β-lactamase enzymes.[4–6]

### PHARMACOKINETICS

The pharmacokinetics of piperacillin and tazobactam are similar. Tazobactam and piperacillin are highly water soluble. They bind very weakly to plasma proteins (20–30%) after intravenous (IV) administration. As both agents leave the intravascular space quickly, distribution is complete within 30 min after administration.[2] Piperacillin and tazobactam are widely distributed in tissues and therapeutic levels are achieved in skin, muscle, intestinal mucosa, appendix tissue, and fatty tissue.[7] The peak concentrations of piperacillin and tazobactam

Address correspondence/reprint requests to Dr. Stephanie M. Culver, Department of Obstetrics and Gynecology, Hennepin County Medical Center, 701 Park Avenue South, Minneapolis, MN 55415-1829.

Received June 24, 1996
Accepted July 8, 1996

in muscle, skin, and gastrointestinal mucosa occur within 1–2 h after infusion.

Piperacillin plasma concentrations, following the infusion of piperacillin/tazobactam, are similar to those attained when equivalent doses of piperacillin are administered alone.[8] The mean peak plasma concentrations are approximately 134, 242, and 298 μg/ml for 2.25-, 3.375-, and 4.5-g doses, respectively. The corresponding mean peak plasma concentrations of tazobactam are 15.24 and 34 μg/ml, respectively.[3] The total clearance decreases slightly with an increase in dose. The plasma half-life of piperacillin and of tazobactam ranges from 0.7 to 1.2 h.[1]

The amount of inhibitor that can be delivered to the site of infection is an important determinant of the efficacy of a drug combination. The standard dose of piperacillin/tazobactam is 3.375 g (3 g of piperacillin and 0.375 g of tazobactam) administered q 6 h.[1] After the dosing of 3 g of piperacillin and 0.375 g of tazobactam over 5 min, the maximum concentration ($C_{max}$) (mg/l) is 336 with an area under the curve (AUC) of 230; $Cl_T$ is 219; and 51.7% is excreted in the urine. Multiple dosing has not been shown to affect the pharmacokinetics.[9] Currently, pharmacokinetic studies are addressing if the doses of 3.375 g q 6 h and 4.5 g q 8 h are bioequivalent. An q-8-h dosing of piperacillin/tazobactam is believed to be possible based on several earlier studies on piperacillin pharmacokinetics in normal patients.

Piperacillin is metabolized to a minimally active desthyl metabolite that insignificantly contributes to the overall elimination of piperacillin. Tazobactam is metabolized to an essentially inactive metabolite (M1).[2] The elimination pathways of both piperacillin and tazobactam include ultrafiltration (kidney); paracellular elimination; and renal, hepatic, and gastrointestinal secretions. Both compounds are believed to be eliminated renally by tubular secretion and glomercular filtration; therefore, renal failure compromises its elimination. It has been suggested that the dosing interval be extended to 8 h in patients with creatinine clearances ($C_{cr}$) that range between 20 and 40 ml/min and to 10 h with $C_{cr}$ of <20 ml/min.[2]

The concomitant administration of probenecid and piperacillin/tazobactam significantly prolongs the β half-life ($t_{1/2bcta}$) for tazobactam and significantly decreases the piperacillin mean $CL_R$.[10] The authors of this study suggested that these changes likely offered no therapeutic advantage or warranted dosage modifications if probenecid is used concurrently.

### SAFETY PROFILE

Overall, piperacillin/tazobactam has a safety profile similar to those of other β-lactam/β-lactamase inhibitor combinations. The adverse reactions, which are generally not severe, rarely interfere with the continuation of therapy.

Only 3.2% of 2,261 patients studied worldwide had adverse effects that warranted the termination of therapy.[1] The most common adverse experiences related to treatment were allergic skin reactions (1.3%) and gastrointestinal disturbances (0.9%), most often diarrhea. Clinical comparative studies have documented a higher incidence of diarrhea with piperacillin/tazobactam treatment than with other combination antibiotic therapies.[11] The most common laboratory abnormalities have been related to hepatic function: a 1.1% increase in total bilirubin levels and a 5.6% increase in alanine aminotransferase levels.[11] The incidence of elevations increased with concurrent aminoglycoside administration.

Piperacillin/tazobactam has been classified as a pregnancy-risk category-B drug.[1]

### SPECTRUM OF ANTIMICROBIAL ACTIVITY—EMPIRIC AND SELECTED

Two large comparative studies were performed to evaluate piperacillin/tazobactam's in vitro antimicrobial activity.[12,13] These studies demonstrated that piperacillin/tazobactam has an excellent empiric spectrum. This combination is highly active against gram-positive bacteria, including strains that produce β-lactamase, and inhibits a wide spectrum of both fastidious and non-fastidious gram-negative bacilli, including *Pseudomonas aeruginosa*. Piperacillin/tazobactam also inhibits a broader spectrum of anaerobes than does ampicillin/sulbactam or ticarcillin/clavulanate.

A large comparative study tested the antimicrobial activity of piperacillin combined with tazobactam at a fixed concentration of 4 μg/ml and a ratio of 8:1. A total of 5,029 clinical isolates of gram-negative and gram-positive aerobic pathogens and 447 fastidious organisms including anaerobes from 5 medical centers were included.[12] The study results were as follows: >95% inhibition of Enterobacteriaceae; 93.5% inhibition for non-enteric gram-nega-

TABLE 1. In vitro activity of broad-spectrum β-Lactams for pelvic infections[15-17a]

| | Susceptibility | | | | | | |
|---|---|---|---|---|---|---|---|
| | Pip | Amp/Sul | Ticar/Clav | Cftax/Cftrx | Cftaz | Imip | Pip/Tazo |
| *Staphylococcus aureus* | 0 | *** | *** | * | * | *** | *** |
| *S. pyogenes* | *** | *** | *** | *** | *** | *** | *** |
| *Enterococcus faecalis* | *** | *** | * | 0 | 0 | ** | *** |
| *Enterobacteriaceae* | *** | * | *** | *** | *** | *** | *** |
| *Neisseria gonorrhoeae* | ** | *** | *** | *** | *** | *** | *** |
| *Pseudomonas aeruginosa* | *** | 0 | * | * | *** | *** | *** |
| *Bacteroides fragilis* | ** | *** | *** | */0 | 0 | *** | *** |
| *B. bivius* | ** | *** | *** | 0 0 | 0 | *** | *** |

[a]Although a useful guide, in vitro activity does not necessarily correlate with clinical response. Pip = piperacillin; Amp/Sul = ampicillin/sulbactam; Ticar/Clav = ticarcillin/clavulanate; Cftax/Cftrx = cefotaxime/ceftriaxone; Cftax = cefmetazole; Imip = imipenem; Pip/Tazo = piperacillin;/tazobactam. Susceptibility: 0 = <50%; * = 50–75%; ** = 75–90%; *** = >90%.

tive bacilli (the most active agent); 89.3% inhibition against staphylococci (ampillicin/sulbactam was the most active agent at 95%); 91% inhibition against enterococci; 93.5% inhibition of all aerobic isolates; 100% of all fastidious isolates tested; and 99.5% inhibition of anaerobic isolates.

A national survey of the in vitro spectrum of piperacillin/tazobactam at 236 medical centers also demonstrated its wide antimicrobial activity.[13] Piperacillin/tazobactam and imipenem displayed the widest antimicrobial spectra, inhibiting >90% of all isolates tested.

Piperacillin/tazobactam, similar to other combination regimens comprising the penicillin group, does not cover methicillin-resistant staphylococci, strains of enterococci that are resistant to both β-lactams and chromosomally mediated β-lactamases.[14] *Pseudomonas* spp. should be covered with higher doses of piperacillin/tazobactam (4/0.5 g q 6 h or 3/0.75 g q 4 h) plus an aminoglycoside.

In summary, in vitro studies have demonstrated the broad antimicrobial activity and clinical efficacy of piperacillin/tazobactam. Table 1 summarizes the in vitro activity of broad-spectrum β-lactams in pelvic infections.[15-17]

### CLINICAL APPLICATIONS

Currently, the Food and Drug Administration (FDA) has approved piperacillin/tazobactam for the treatment of intraabdominal infections, skin and skin-structure infections, postpartum endometritis or pelvic inflammatory disease (PID), and community- and hospital-acquired pneumonia.[1]

The majority of pelvic infections are polymicrobial in nature, with mixed aerobic, facultative, and anaerobic bacteria involved. The medical literature supports empiric treatment regimens with adequate coverage comparable to a single-agent cephalosporin or extended-spectrum penicillins with high rates of cure. In previous studies, piperacillin alone has been shown to be as efficacious as standard regimens in the treatment of pelvic infections.[18,19]

TABLE 2. Example costs of acquisition and IV administration of various antibiotic regimens

| Antibiotic | Dosing | Cost/day |
|---|---|---|
| Ampicillin/sulbactam | 3 g q 6 h | $59.68 |
| Ticarcillin/clavulanate | 3.1 g q 6 h | $58.96 |
| Piperacillin/tazobactam | 3.375 g q 6 h | $65.27 |
| | 4.5 g q 8 h | $60.09 |
| Imipenem/cilastatin | 500 mg q 8 h | $95.05 |
| Cefmetazole | 2 g q 8 h | $51.24 |
| Cefotaxime | 2 g q 8 h | $58.93 |
| Clindamycin/gentamicin | 900 mg/80 mg q 8 h | $37.57 |

In a randomized study by Sweet et al.,[20] piperacillin and tazobactam, 3.375 g q 6 h (N = 196) was compared with clindamycin, 900 mg, and gentamicin, 2.5–5.0 mg/kg/d q 8 h (N = 103) in hospitalized women with pelvic infections. A total of 87% of the infections were either endometritis or PID. The combination of piperacillin/tazobactam produced favorable clinical responses in 85% of the patients vs. 87% of the gentamicin/clindamycin-treated group. These differences were not clinically significant. The bacteriologic responses were comparable in the 2 groups: 78% and 82%, respectively. Some *Streptococcus* spp. were the only organisms isolated that were resistant to piperacillin/tazobactam. The investigators concluded that the combination of

TABLE 3. Activity of piperacillin/tazobactam against major pathogens

| Examples of organisms with $MIC_{90}$ | | |
|---|---|---|
| 1≤μg/ml | 2–8 μg/ml | 16–64 μg/ml |
| *Streptococcus pyogenes* | *Staphylococcus aureus* (oxacillin-susceptible) | |
| *S. agalactaiae* | | |
| *S. pneumoniae* | *S. aureus*, coagulase-negative (oxacillin-susceptible) | |
| *S. viridans* | | |
| Groups C, D, F, G streptococci | *Enterococcus faecalis* | |
| | | *Escherichia coli* |
| *Haemophilus influenzae* | *Klebsiella pneumoniae* | *Pseudomonas aeruginosa* |
| *Moraxella catarrhalis* | *K. oxytoca* | *Aeianetobacter anitratus* |
| | *Proteus mirabilis* | *Enterobacter cloacae* |
| | *Serratia marcescens* | *E. aerogens* |
| *Anaerobic gram-positive cocci* | *Bacteroides fragilis* | *B. fragilis* group |
| | *B.* non-*fragilis* group | |

piperacillin/tazobactam is an effective, well-tolerated regimen.

Two non-comparative studies of piperacillin/tazobactam (4/0.5 g q 8 h) for the treatment of pelvic infections (mean duration of 5 days) are in the current literature. Tapp et al.[21] used piperacillin/tazobactam in a non-comparative study to treat 25 patients with pelvic infections. Of the 20 evaluable patients, 95% were clinically cured. Six patients had organisms isolated, none of which was resistant to piperacillin or the piperacillin/tazobactam combination. Sanders[22] studied patients diagnosed with pelvic infections (salpingitis, endometritis, tubo-ovarian abscesses, cuff infections, and pelvic soft-tissue infections). Eighty-nine patients (86%) completed the study, of whom 66% were clinically evaluable and 29% microbiologically evaluable. Ninety percent of the patients demonstrated favorable clinical outcomes by the study's end point. Bacteriologic eradication was achieved in 90% of these patients.

A direct comparison of ticarcillin/clavulanate and piperacillin/tazobactam for the treatment of lower-respiratory-tract infections showed piperacillin/tazobactam to be clinically superior. In the treatment of skin and soft-tissue infections, the 2 regimens were comparable. Piperacillin/tazobactam was at least as effective as imipenem/cilastatin and clindamycin plus gentamicin for the treatment of intraabdominal infections, including the q-8-h dosing regimen.[14]

Piperacillin alone for prophylaxis in preterm membrane rupture has been studied. A double-blind, placebo-controlled trial demonstrated that the use of IV piperacillin for 72 h in preterm premature rupture of the membranes significantly prolonged the latency period between membrane rupture and delivery.[23] The mean latency period was prolonged in the piperacillin group vs. the control group (11.4 ± 18.8 vs. 6.1 ± 13.6 days, respectively; $P = 0.001$).

### COST

Although the costs for the acquisition and administration of antibiotics vary by institution, the relative costs are usually comparable. The costs for acquisition plus a $5.00/dose charge for IV preparation and administration for various antibiotic regimens commonly utilized for the treatment of obstetric and gynecologic infections at our hospital are given in Table 2.

Therefore, piperacillin/tazobactam appears to be comparable to other β-lactamase inhibitor combinations and much less expensive than imipenem-cilastatin. Although single-agent cephalosporins and clindamycin/gentamicin are less expensive, their enterococcal coverage is lacking. While the significance of enterococcal coverage is still under debate, the recent increase in vancomycin-resistant enterococci is of great concern, indicating that enterococcal coverage may become a factor in the selection of empiric antibiotics in the future.

### CONCLUSIONS

Piperacillin/tazobactam is an appropriate regimen for the treatment of gynecologic infections. This regimen covers a broad spectrum of pathogens, thereby providing empiric coverage (Table 3). Piperacillin/tazobactam has clinical and bacteriologic responses comparable to clindamycin/gentamicin, demonstrating efficacy in polymicrobial infections, good penetration of gynecologic tissues, and good tolerance.

### REFERENCES

1. Package Information. Zosyn (Piperacillin/Tazobactam) Carolina, Puerto Rico, February 1995.
2. Sörgel F, Kinzig M: The chemistry, pharmacokinetics and tissue distribution of piperacillin/tazobactam. J Antimicrob Chemother 31(Suppl A):39–60, 1993.
3. Higashitani F, Hyodo A, Ishida N, Inoue M, Mitsashashi S: Inhibition of β-lactamases by tazobactam and in-vitro antibacterial activity of tazobactam combined with piperacillin. J Antimicrob Chemother 25:567–574, 1990.
4. Weler DA, Sanders CC: Diverse potential of β-lactamase inhibitors to induce class I enzymes. Antimicrob Agents Chemother 34:156–158, 1990.
5. Stobberingh EE: In vitro effect of YTR 830 (tazobactam) in plasmid and chromosomally mediated β-lactamases. Chemotherapy 36:209–214, 1990.
6. Akova M, Yang Y, Livermore DM: Interactions of tazobactam and clavulanate with inducible and constitutively expressed class β-lactamases. J Antimicrob Chemother 25:199–208, 1990.
7. Kinzig M, Sörgel F, Brismar Band Nord CE: Pharmacokinetics and tissue penetration of tazobactam and piperacillin in patients undergoing colorectal surgery. Antimicrob Agents Chemother 36:1997–2004, 1992.
8. Wise R, Logan M, Cooper M, Andreas JM: Pharmacokinetics and tissue penetration of tazobactam administered alone and with piperacillin. Antimicrob Agents Chemother 35:108–114, 1991.
9. Rohr M, Böckmann U, Endrigkert S, Schütz K, Nier H: Antibiotic therapy and perioperative prophylaxis in acute appendicitis with piperacillin/tazobactam (abstract 1435). Proceedings of the 18th International Congress of Chemotherapy, Stockholm, June 27–July 2, 1993, p 362.
10. Ganes D, Batra V, Faulkner R, et al.: Effect of probenecid on the pharmacokinetics of piperacillin and tazobactam in healthy volunteers. Pharm Res 8:5–299, 1991.
11. Schoonover LL, Occhipinti DJ, Rodvold K, Danziger LH: Piperacillin/tazobactam: A new β-lactam-lactamase inhibitor combination. Ann Pharmacol 29:501–514, 1995.
12. Marchall SA, Aldrige KE, Allen SD, Fuchs PC, Gerlach HE, Jones RN: Comparative antimicrobial activity of piperacillin/tazobactam tested against more than 5,000 recent clinical isolates in the last five years. Diagn Microbiol Infect Dis 21:153–168, 1995.
13. Baron EJ, Jones RN: National survey of the in vitro spectrum of piperacillin/tazobactam tested against more than 40,000 aerobic clinical isolates from 236 medical centers. Diagn Microbiol Infect Dis 21:141–150, 1995.
14. Sanders WE Jr, Sanders CC: Piperacillin/tazobactam: A critical review of the evolving clinical literature. Clin Infect Dis 22:107–112, 1996.
15. File TM Jr, Tan JS: Efficacy and safety of piperacillin/tazobactam in skin and soft tissue infections. Eur J Surg 573(Suppl):51–55, 1994.
16. Faro S: Infectious disease relations to cesarean section. Obstet Gynecol Clin North Am 15(4):685–695, 1988.
17. Jones RN, Pfaller MA, Fuchs PC, Aldridge K, Allen SD, Gerlach EH: Piperacillin/tazobactam (YTR 830) combination. Comparative antimicrobial activity against 5889 recent aerobic clinical isolates and 60 *Bacteroides fragilis* group strains. Diagn Microbiol Infect Dis 12(6):489–494, 1989.
18. Gilstrap LC, Maier RC, Gibbs RS, Connec SL, Clair PJ: Piperacillin versus clindamycin plus gentamicin for pelvic infections. Obstet Gynecol 64:1762–1765, 1984.
19. Sweet RL, Robbie MO, Ohm-Smith M, Hadley WK: Comparative study of piperacillin versus cefoxitin in the treatment of obstetric and gynecologic infections. Am J Obstet Gynecol 145:342–349, 1983.
20. Sweet RL, Roy S, Faro S, et al.: Piperacillin and tazobactam versus clindamycin and gentamicin in the treatment of hospitalized women with pelvic infection. Obstet Gynecol 83:280–286, 1994.
21. Tapp A, Wise B, Cordozo L: Efficacy and safety of piperacillin/tazobactam in gynecological infection. Infect Med 10(Suppl A):61–64, 1993.
22. Sanders CV: Treatment of polymicrobial gynecologic and skin and skin structure infections: World-wide clinical trials. Infect Dis Clin Pract 4(Suppl), 1995.
23. Lockwood CJ, et al.: Double-blind, placebo-control trial of piperacillin prophylaxis in preterm membrane rupture. Am J Obstet Gynecol 169:970, 1993.
